# Extraction-Free Absolute Quantification of Circulating miRNAs by Chip-Based Digital PCR

**DOI:** 10.3390/biomedicines10061354

**Published:** 2022-06-08

**Authors:** Yuri D’Alessandra, Vincenza Valerio, Donato Moschetta, Ilaria Massaiu, Michele Bozzi, Maddalena Conte, Valentina Parisi, Michele Ciccarelli, Dario Leosco, Veronika A. Myasoedova, Paolo Poggio

**Affiliations:** 1 Centro Cardiologico Monzino IRCCS, 20138 Milan, Italy; yuri.dalessandra@bio-techne.com (Y.D.); vincenza.valerio@cardiologicomonzino.it (V.V.); donato.moschetta@cardiologicomonzino.it (D.M.); ilaria.massaiu@cardiologicomonzino.it (I.M.); michele.bozzi@cardiologicomonzino.it (M.B.); veronika.myasoedova@cardiologicomonzino.it (V.A.M.); 2 Dipartimento di Scienze Farmacologiche e Biomolecolari, Università degli Studi di Milano, 20122 Milan, Italy; 3 Department of Translational Medical Sciences, University of Naples Federico II, 80138 Naples, Italy; maddalena-conte@libero.it (M.C.); valentina.parisi@unina.it (V.P.); dleosco@unina.it (D.L.); 4 Casa di Cura San Michele, 81024 Maddaloni, Italy; 5 Department of Medicine, Surgery and Dentistry, University of Salerno, 84084 Fisciano, Italy; mciccarelli@unisa.it

**Keywords:** microRNA, digital PCR, biomarkers, extraction-free, plasma

## Abstract

Circulating microRNAs (miRNA) have been proposed as specific biomarkers for several diseases. Quantitative Real-Time PCR (RT-qPCR) is the gold standard technique currently used to evaluate miRNAs expression from different sources. In the last few years, digital PCR (dPCR) emerged as a complementary and accurate detection method. When dealing with gene expression, the first and most delicate step is nucleic-acid isolation. However, all currently available protocols for RNA extraction suffer from the variable loss of RNA species due to the chemicals and number of steps involved, from sample lysis to nucleic acid elution. Here, we evaluated a new process for the detection of circulating miRNAs, consisting of sample lysis followed by direct evaluation by dPCR in plasma from healthy donors and in the cardiovascular setting. Our results showed that dPCR is able to detect, with high accuracy, low-copy-number as well as highly expressed miRNAs in human plasma samples without the need for RNA extraction. Moreover, we assessed a known myocardial infarction-related miR-133a in acute myocardial infarct patients vs. healthy subjects. In conclusion, our results show the suitability of the extraction-free quantification of circulating miRNAs as disease markers by direct dPCR.

## 1. Introduction

MiRNAs are small endogenous non-coding RNAs involved in the regulation of gene expression by the modulation of messenger RNAs (mRNAs) translation and stability. They influence vital cellular processes such as responses to external stimuli, proliferation, differentiation, and apoptosis [1], and their dysregulation is associated with various pathologies [2]. In recent years, miRNAs emerged as possible specific biomarkers of several conditions because of their stable expression in almost all body fluids and due to the development of accurate and quantitative techniques for their detection [3]. In particular, several groups investigated the potential use of circulating miRNAs in the diagnostic and/or prognostic setting of cardiovascular diseases in order to formulate tailored therapeutic approaches [4]. However, the use of circulating miRNAs as biomarkers can be hypothesized only if reliable and accurate methods for quantification are devised. Quantitative reverse transcription PCR (RT-qPCR) represents, to date, the method of choice for nucleic acid quantification. Nevertheless, one of the major drawbacks of this technique is the normalization issue. Indeed, there is a lack of consensus about both normalization methods and the choice of reliable endogenous reference miRNAs [5,6]. Digital PCR (dPCR) is a recent technology that allows nucleic acid measurement based on the segregation of the sample into thousands of separate micro-reactions of defined volume [7]. After PCR, each partition is analyzed for amplicon presence/absence, and the number of molecules in each reaction is estimated by applying the Poisson distribution [8]. When compared to RT-qPCR, the dPCR technique was described as being able to perform absolute quantification, thus bypassing normalization issues [9,10,11]. Moreover, when coping with low amounts of nucleic acids, it presents higher precision [12] and sensitivity [13], possibly because of its increased resistance to PCR inhibitors [14].

Interestingly, several works comparing dPCR performance with RT-qPCR in evaluating circulating miRNAs expression indicated an almost equal sensitivity for the two methods. At the same time, though, there was an almost undisputed consensus about the superior accuracy and precision of dPCR [15,16,17]. In addition to detection efficiency, one of the most important steps in mRNA (and miRNA) expression experiments is nucleic acid extraction. This step is usually conducted by means of specific kits and/or chemicals, and the selection of one specific method can greatly affect the following results [18,19,20,21].

In the present study, we assessed the reliability of chip-based dPCR in detecting circulating miRNAs with high accuracy and precision by evaluating six differently expressed plasma miRNAs, which we previously found to have low, medium, and high expression levels [22], in samples from healthy subjects. In addition, we tested if chip-based dPCR could be applied to a clinical setting evaluating miR-133a in non-ST-segment-elevation-myocardial-infarction (NSTEMI) patients, previously found to be upregulated upon myocardial infarction in human plasma samples [23,24].

## 2. Materials and Methods

### 2.1. Human Specimens

The Institutional Review Board and the Ethical Committee of Centro Cardiologico Monzino IRCCS (university hospital) approved this study (CCM 1068). The investigation conformed to the principles outlined in the Declaration of Helsinki (1964).

Peripheral blood samples were collected into EDTA-coated tubes (Vacutainer Systems, Becton Dickinson, Franklin Lakes, NJ, USA), kept on ice, and centrifuged at 3000× *g* for 10 min at 4 °C within 30 min after being drawn. Plasma was separated, centrifuged again to precipitate remaining cells, aliquoted, and stored at −80 °C until analyses were performed.

### 2.2. RNA Extraction and miRNA Reverse Transcription

Total RNA extraction was performed from 200 µL of plasma/sample using the Total RNA Purification Plus Kit (Norgen Biotek Corp., Thorold, ON, USA). Final elution was performed using 50 µL of Elution Solution A, as indicated by the manufacturer’s protocol. In the case of the “no-extraction” protocol, 200 µL of plasma from the same samples were incubated at room temperature with proteinase K (200 µg/mL, final concentration) for 15 min with occasional flicking, followed by 5 min at 75 °C in agitation.

Since the quantification of circulating miRNAs is not possible due to the technological limitations of actual tools, 2 μL of RNA from each sample were used for two-step PCR amplification with the TaqMan Advanced miRNA cDNA Synthesis Kit (Thermo Fisher Scientific, Waltham, MA, USA; A28007) following the manufacturer’s instructions, regardless of “extraction” or “no-extraction” protocols. In brief, we followed the described steps without modifications. (1) Poly(a) tailing addition: polyadenylation at 37 °C for 45 min followed by incubation at 65 °C to stop the reaction. (2) 5′-end Adaptor ligation: 16 °C for 1 h. (3) Reverse transcription (RT): 42 °C for 15 min, using a universal RT primer included in the kit, followed by incubation at 85 °C for 5 min to stop the reaction. (4) miRNAs universal pre-amplification (using proprietary primers included in the kit): enzyme activation at 95 °C for 5 min, denaturation at 95 °C for 3 s and annealing/extension at 60 °C for 30 s (14 cycles), and finally, incubation at 99 °C for 10 min to stop the reaction. cDNA samples were stored at −80 °C until PCR further analyses. A synthetic miRNA from *C. elegans*, cel-miR-54-3p, which is not present in human samples, was used as a spike-in control to assess dPCR efficiency.

### 2.3. Chip-Based Digital PCR

Chip-based dPCR was performed on a QuantStudio 3D Digital PCR System platform composed of the QuantStudio 3D Instrument (Thermo Fisher Scientific; 4489084), the Dual Flat Block GeneAmp PCR System 9700 (Thermo Fisher Scientific; 4484078), and the QuantStudio 3D Digital PCR Chip Loader (Thermo Fisher Scientific; 4482592). dPCR was performed according to the manufacturer’s instructions. In particular, enzyme activation was performed at 96 °C for 10 min, denaturation at 98 °C for 30 s, followed by annealing/extension at 56 °C for 2 min (40 cycles), and then final extension at 60 °C for 2 min. Analysis was executed with the online version of the QuantStudio 3D AnalysisSuite (Thermo Fisher Cloud, Waltham, MA, USA). The evaluated copy numbers for each miRNA are expressed as the mean of Log10 of the copy number/µL within a 95% confidence interval (CI). Upper and lower limits of the 95% CI are indicated within square brackets.

Primers and probes purchased from Thermo Fisher Scientific were labelled with FAM dyes and used to evaluate the expression of candidate miRNAs (Supplementary Table S1).

### 2.4. Statistical Analysis

We compared each gene expression technique using a correlation analysis: Pearson’s correlation coefficient (R), the R^2^ coefficient, and the *p*-value were computed. We performed this kind of analysis on the averaged expression values across all samples, and each value was previously “mean-centered”. The analysis in the clinical setting, comparing healthy subjects and cardiovascular disease patients, was performed using the Student’s *t*-test.

## 3. Results

### 3.1. Assessment of dPCR Efficiency

**Figure 1:**
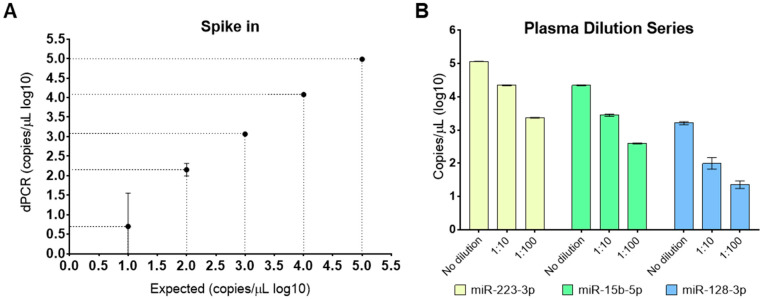
Digital PCR capability to detect miRNA in a wide range of concentrations. (**A**) Regression analysis between expected (x-axis) and dPCR-assessed (y-axis) expression of serial dilutions (10^5^ to 10^1^ copies/µL) of synthetic cel-miR-54-3p in triplicate. The data are expressed as Log10 (miRNA copies/µL of the sample), and the bars represent the 95% confidence interval. (**B**) Expression levels of undiluted, 1:10, and 1:100 dilutions of miR-223-3p, miR-15b-5p, and miR-128-3p in one healthy subject in triplicate. No RNA extraction was performed on the plasma sample. The data are expressed as Log10(miRNA copies/µL of the sample), and error bars represent the 95% confidence interval.

As a first step, we assessed dPCR efficiency in detecting a synthetic miRNA designed from nematode *C. elegans* cel-miR-54-3p at different dilutions (10^5^ to 10^1^ copies/µL). Linear regression analysis between expected and assessed copy numbers for miR-54-3p showed an R^2^ of 0.999 (*p* < 0.0001; Figure 1A), thus indicating the suitability of dPCR in assessing with precision the expression of selected miRNAs, even in a “very-low abundance” setting. Next, we assessed whether extraction-free RNA from human plasma could be used for direct miRNA detection by dPCR. To this aim, we analyzed miRNA expression in undiluted, 1:10, and 1:100 dilutions of “no extraction” plasma samples. Three miRNAs were selected (miR-223-3p, miR-15b-5p, and miR-128-3p) because of their high, intermediate, and low expression levels in human plasma based on our previous experiences (not shown). Our experiments showed that all miRNAs were detectable regardless of dilution conditions by dPCR (Figure 1B).

### 3.2. Optimization of dPCR Conditions for Plasma miRNAs Detection in the Absence of RNA Extraction

**Figure 2:**
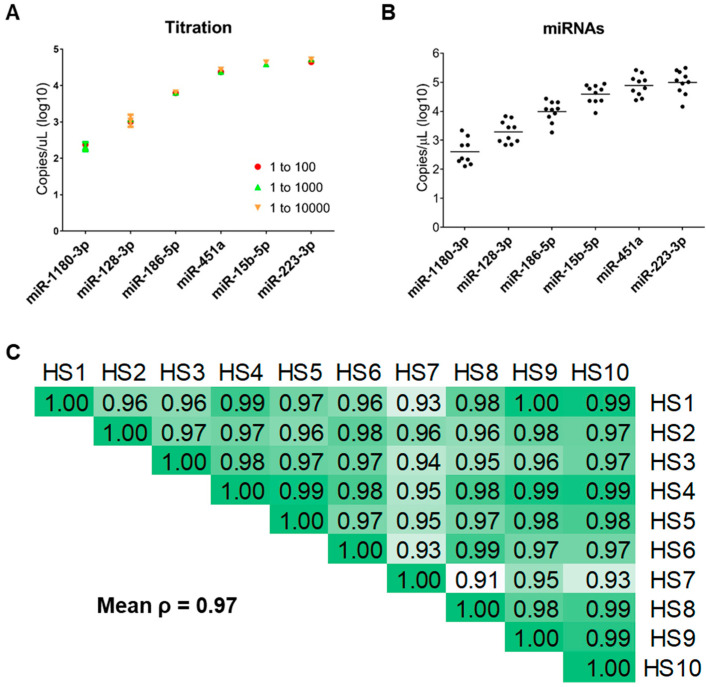
Digital PCR settings for miRNA detection in human plasma without RNA extraction. (**A**) Expression levels of miR-1180-3p, miR-128-3p, miR-186-5p, miR-451a, miR-15b-5p, and miR-223-5p at different dilutions in a plasma sample without RNA extraction. The data are expressed as Log10 (miRNA copies/µL of the sample), and error bars represent the 95% confidence interval. Each color and shape indicate the different dilution conditions. (**B**) Dot plot depicting the expression levels of miR-1180-3p, miR-128-3p, miR-186-5p, miR-451a, miR-15b-5p, and miR-223-5p in ten plasma samples without RNA extraction. The data are expressed as Log10 (miRNA copies/µL of the sample). Horizontal bars represent mean values. (**C**) Correlation matrix conducted on the expression of levels of miR-1180-3p, miR-128-3p, miR-186-5p, miR-451a, miR-15b-5p, and miR-223-5p in no-extraction samples from ten healthy subjects (HS). Green-shade intensity is directly proportional to the mean Pearson correlation coefficient.

The process of miRNAs expression assessment involves an amplification step (preAmp) after the conversion of RNA to cDNA, followed by dilution. Since we did not perform RNA extraction, we decided to evaluate whether different dilution settings could affect miRNA detection. Thus, after assessing the suitability of undiluted “no extraction” plasma miRNAs for dPCR, we investigated the detection of plasma miRNAs usually presenting expression levels ranging from low to high in standard conditions. Thus, we evaluated the copy number/µL of six different circulating miRNAs (miR-1180-3p, miR-128-3p, miR-186-5p, miR-451a, miR-15b-5p, and miR-223-3p). Different serial preAmp dilutions for each miRNA (1:100, 1:1000, and 1:10,000) were prepared in order to identify those most suitable for each specific target. As depicted in Figure 2A, our results showed very high overlap in terms of copies/µL, regardless of dilution, for all evaluated miRNAs. Given its low expression, miR-1180-3p was not tested beyond the 1:1000 dilution. The selected working conditions, based on chip quality (i.e., threshold = 0.6; Supplementary Figure S1) and precision (the lowest value; Supplementary Table S2), for each miRNA were identified as: 1:100 for miR-186-5p, miR-128-3p, miR-451a, and miR-1180-3p; 1:1000 for miR-15b-5p and miR-223-5p.

Following the evaluation of dPCR-mediated detection of extraction-free plasma miRNAs, we investigated whether we could obtain consistent results in terms of reproducibility. Hence, we assessed the expression of miR-1180-3p, miR-128-3p, miR-186-5p, miR-451a, miR-15b-5p, and miR-223-5p in plasma samples from 10 healthy donors. As shown in Figure 2B, we obtained good results in terms of reproducibility and consistency for all miRNAs, none of which presented a widely sparse distribution. Consistently with previous results, miR-1180-3p showed the lowest expression, while miR-223-5p was the one with the highest copy number/µL. Further analyses conducted on all evaluated miRNAs indicated a very good correlation between the expression levels of all miRNAs in all samples, with a mean Pearson coefficient of 0.97 (Figure 2C).

To further corroborate our analyses, we evaluated the consistency of our detection method upon three different technical replicates executed at different times. We focused on the two low-expression miRNAs, namely miR-1180-3p and -186-5p, in five no-RNA-extraction samples upon three distinct dPCR runs conducted on different days. The results were highly reproducible for both miRNAs, with miR-186-5p presenting a mean coefficient of variation (CV) of 3.1%, while miR-1180-3p demonstrated an even higher accuracy, with a mean CV of 2.9% for the three runs (Supplementary Table S3).

### 3.3. Evaluation of RNA Extraction Influence on Plasma miRNA Expression

**Figure 3:**
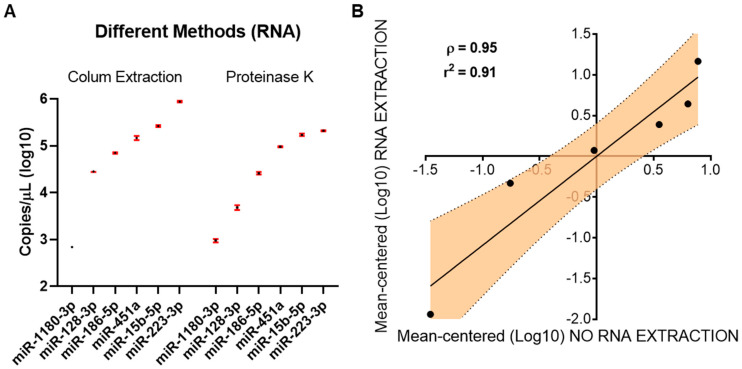
Comparison of column-based RNA extraction (Colum Extraction) or no-extraction (Proteinase K) protocols. (**A**) Normalized copies of column-based RNA extraction and no-extraction protocols on starting volume (50 µL and 200 µL, respectively). The level of each miRNA was calculated as Log10 of copy number/µL. Black dots represent the mean value, while error bars (red) represent the 95% confidence interval. (**B**) Correlation analysis between plasma miRNA expression after either column-based RNA extraction and no-extraction protocols. The level of each miRNA was calculated as Log10 of copy number/µL and reported as mean-centered.

RNA-based studies usually involve nucleic acid extraction from a specific substrate as a starting point. Total RNA extraction can be achieved by means of different methods, which can consist of lysis followed by either nucleic acid precipitation or binding to affinity columns or magnetic beads. Each of the listed methods presents advantages and disadvantages, but all have different impacts on the final composition of the RNA “populations” obtained. Since column-based extraction is one of the most adopted approaches in the case of liquid samples, we decided to compare its outcome in terms of plasma miRNA expression with the “no-extraction” approach (proteinase K). In particular, we assessed the plasma expression of miR-1180-3p, miR-128-3p, miR-186-5p, miR-451a, miR-15b-5p, and miR-223-5p by dPCR in a plasma sample obtained from a healthy subject undergoing either column-based extraction or “no-extraction” protocol. Figure 3A depict the results in terms of Log10 of copy number/µL for all evaluated miRNAs. Both “conditions” showed the same hierarchic order in terms of miRNAs expression (ordered from lowest to highest expression), although some discrepancies seem to present. However, the difference in terms of yield when looking at the detected levels of miRNAs upon column-based RNA extraction vs. the no-extraction protocol is negligible. Indeed, the mean miRNAs Log10 expression ratio between the two protocols is 1.08, indicating a good relationship between the two approaches. However, two miRNAs, namely miR-128-3p and miR-223-3p, showed slightly different results when comparing the two protocols with a ratio of 1.21 and 1.12, respectively. Nevertheless, we observed a very good correlation (*p* = 0.003) between the results obtained in terms of expression from the two protocols (Figure 3B).

### 3.4. No-Extraction dPCR-Based miRNA Detection in the Clinical Setting

**Figure 4:**
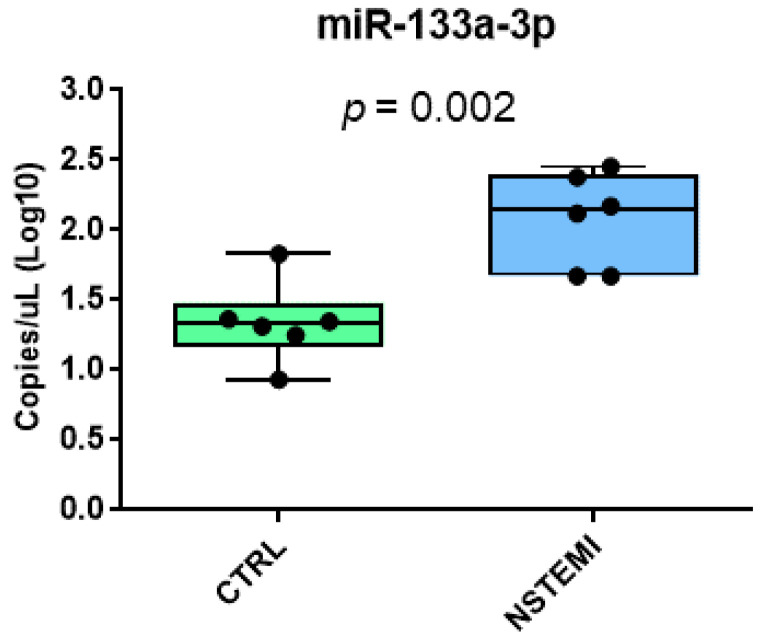
Plasma miRNA evaluation in a clinical setting. Plasma miR-133a-3p expression in healthy subjects (CTRL) and non-ST-segment-elevation-myocardial-infarction (NSTEMI) patients employing the proteinase K protocol. The level of the miRNA was calculated as Log10 of copy number/µL.

After demonstrating the suitability of dPCR to detect even low-abundance circulating miRNAs in healthy subjects, we decided to challenge the “clinical arena”. Thus, we investigated the expression levels of cardiac disease-related miR-133a-3p in no-RNA-extraction plasma of NSTEMI patients (*n* = 6) in comparison to healthy subjects (*n* = 6). As shown in Figure 4, in keeping with the literature, our data demonstrated a higher expression for miR-133-3p in NSTEMI patients (2.07 [2.02, 2.13] copies/µL) vs. controls (1.33 [1.21, 1.46] copies/µL, *p* = 0.002).

## 4. Discussion

This is, to the best of our knowledge, the first work reporting on the extraction-free detection of circulating miRNAs by dPCR. We think that our data can lead to at least two important conclusions. The first is that we were able to demonstrate that the extraction-free approach is viable and valid for dPCR even in a context where the quantity of miRNAs present in the substrate is very low, as in the case of plasma. The second relies on the fact that dPCR is a suitable tool for the direct and rapid quantification of circulating miRNAs in the clinical setting. Indeed, previous studies already demonstrated the suitability of dPCR to assess the modulation of circulating miRNAs and their possible use as biomarkers in the disease context [25,26].

A recent work by Su and colleagues [27] described the application of an extraction-free method to assess the expression of circulating miRNAs by “canonical” RT-qPCR in coronary artery disease. However, it was demonstrated that droplet-digital PCR has greater precision and improved day-to-day reproducibility over RT-qPCR with similar sensitivity [15]. In addition, the end-point approach employed by dPCR could offer better performances being less prone to suffer from differences in sample quality and more resilient to PCR inhibitors [15].

In keeping with previous literature, we showed that RNA extraction could be avoided without affecting the sensitivity of dPCR in detecting even the lowest-abundant miRNAs. Indeed, our data clearly indicate that, upon a proper assessment of the best conditions for each miRNA, dPCR is a suitable tool to analyze the expression of poorly expressed miRNAs in plasma samples even without RNA extraction. We also observed a negligible overall difference in terms of yield when looking at the detected levels of miRNAs upon column-based RNA extraction vs. the no-extraction protocol. Nevertheless, two miRNAs showed slightly different results when comparing the two protocols. This discrepancy could be explained by a different “compartmentalization” of the analyzed miRNAs in the plasma. Indeed, the use of proteinase K in the no-extraction protocol led to the release of protein-bound miRNAs but could be less effective on other plasma-miRNA vehicles, such as extracellular vesicles and lipoproteins and other known transporters [28]. However, an advantage of avoiding the extraction steps is represented by the reduction in terms of expense (i.e., not using extraction reagents or RNA affinity columns, which adds to the total cost of miRNA evaluation) and working time (i.e., jumping directly to the PCR mix step saving about 30 min per batch). It is clear that dPCR is unquestionably far from being a high throughput method. Nevertheless, the combination of absolute quantitation with high reproducibility, without the generation of a standard curve, and lack of extraction could allow the detection of miRNAs by dPCR even in the clinical setting.

We must also consider that the emerging role of miRNAs as potential biomarkers for clinical applications requires the standardization of miRNA processing [29]. To date, the most controversial issue related to miRNA level quantification is represented by the normalization step since it is well known that this step could greatly influence the results [29]. Our data indicate that the evaluation of miRNA levels in human plasma via direct and absolute dPCR quantification could be obtained without a normalizer. Indeed, we could see a significant increment in miR-133a-3p levels in plasma obtained from NSTEMI patients without normalizing it to any other miRNA, exogenous or endogenous [23,24]. In addition, it was shown that in NSTEMI patients, miR-133a-3p was only detected in serum and not in plasma by RT-qPCR, indicating a different pattern of circulating miRNA expression in these patients [30]. However, our data, showing the detection of this specific miRNA in the plasma of NSTEMI patients, corroborate the hypothesis that the use of a high precision technique (i.e., dPCR) could be implemented in clinical laboratory analyses.

A major limitation of our study is represented by the small sample size while setting up dilution conditions pre-, post-RT, pre-amp, and validation. However, we must point out that on many occasions, different conditions led to overlapping results and that inter-day reproducibility was very high. In addition, once characterization was finished, we were able to assess the expression of several miRNAs in plasma both with and without extraction with success and covering results.

## 5. Conclusions

Our results showed that dPCR is able to detect, with high accuracy, low-copy-number as well as highly expressed miRNAs in human plasma samples without the need for RNA extraction. Moreover, the ability to assess a known myocardial infarction-related miRNA in acute myocardial infarct patients vs. healthy subjects without the use of a normalizer encourages the use of this high precision technique in the clinical setting. In conclusion, our results show the suitability of extraction-free quantification of circulating miRNAs as disease markers by direct dPCR.

## Data Availability

Data generated for this study are available upon reasonable request to the corresponding author P.P. (paolo.poggio@cardiologicomonzino.it).

## Supplementary Materials

The following supporting information can be downloaded at: https://www.mdpi.com/article/10.3390/biomedicines10061354/s1, Supplementary Figure S1: miRNA detection in human plasma by digital PCR; Supplementary Table S1: miRNA used with respective mature sequence and manufacturer codes; Supplementary Table S2: Digital PCR settings for miRNA detection in human plasma without RNA extraction; Supplementary Table S3: Detection of expression levels of low-abundance miR-186-5p, and miR-1180-3p is highly consistent at different times across multiple samples.
