# Supplementary material for: Extraction-Free Absolute Quantification of Circulating miRNAs by Chip-Based Digital PCR
Source: Biomedicines. 2022 Jun 8;10(6):1354. doi: 10.3390/biomedicines10061354 (PMC9220272; doi:10.3390/biomedicines10061354)
Supplement: Supplementary file 1 [file biomedicines-10-01354-s001.zip › biomedicines-1752953-supplementary.pdf]

DATA SUPPLEMENT

Extraction-free absolute quantification of circulating miRNAs by chip-based digital PCR

Yuri D'Alessandra[1], Vincenza Valerio[1], Donato Moschetta[1,2], Ilaria Massaiu[1], Michele Bozzi[1], Maddalena Conte[3,4], Valentina Parisi[3], Michele Ciccarelli[5], Dario Leosco[3], Veronika A Myasoedova[1], Paolo Poggio[1,*]

[1] *Centro Cardiologico Monzino IRCCS, Milan, Italy*

[2] *Università degli Studi di Milano, Dipartimento di Scienze Farmacologiche e Biomolecolari, Milan, Italy*

[3] *University of Naples Federico II, Department of Translational Medical Sciences, Naples, Italy*

[4] *Casa di Cura San Michele, Maddaloni, Italy*

[5] *University of Salerno, Department of Medicine, Surgery and Dentistry, Fisciano, Campania, Italy*

* *Correspondence*

Paolo Poggio, Ph.D., Head of the Unit for the Study of Aortic, Valvular and Coronary Pathologies, Centro Cardiologico Monzino IRCCS, Via Parea 4, 20138, Milan, Italy.

Telephone: +39 02 5800 2853 Email: paolo.poggio@cardiologicomonzino.it.

SUPPLEMENTARY TABLES

Supplementary Table S1: miRNA used with respective mature sequence and manufacturer codes.

miRNA	Mature miRNA Sequence	Assay ID
cel-miR-54-3p	UACCCGUAAUCUUCAUAAUCCGAG	478410_mir
has-miR-1180-3p	UUUCCGGCUCGCGUGGGUGUGU	477869_mir
has-miR-128-3p	UGGUUCUAGACUUGCCAACUA	477892_mir
has-miR-186-5p	CAAAGAAUUCUCCUUUUGGGCU	477940_mir
has-miR-451a	AAACCGUUACCAUUACUGAGUU	478107_mir
has-miR-15b-5p	UAGCAGCACAUCAUGGUUUACA	478313_mir
has-miR-223-3p	UGUCAGUUUGUCAAAUACCCCA	477983_mir

Supplementary Table S2: Digital PCR settings for miRNA detection in human plasma without RNA extraction.

miRNA	1 to 100			
	Log Copies/uL	Log 95% CI MAX	Log 95% CI MIN	Precision
**miR-15b-5p**				
**miR-186-5p**	3.794	3.806	3.787	2.08%
**miR-128-3p**	3.000	3.016	2.981	4.29%
**miR-451a**	4.371	4.381	4.362	2.29%
**miR-223-3p**	4.644	4.665	4.622	5.07%
**miR-1180-3p**	2.374	2.409	2.338	8.50%
**miRNA**	**1 to 1000**			
	**Log Copies/uL**	**Log 95% CI MAX**	**Log 95% CI MIN**	**Precision**
**miR-15b-5p**	4.588	4.598	4.578	2.40%
**miR-186-5p**	3.809	3.834	3.791	5.25%
**miR-128-3p**	3.074	3.123	3.025	11.92%
**miR-451a**	4.380	4.392	4.368	2.81%
**miR-223-3p**	4.721	4.731	4.712	2.13%
**miR-1180-3p**	2.320	2.439	2.201	31.58%
**miRNA**	**1 to 10000**			
	**Log Copies/uL**	**Log 95% CI MAX**	**Log 95% CI MIN**	**Precision**
**miR-15b-5p**	4.632	4.659	4.606	6.24%
**miR-186-5p**	3.809	3.881	3.738	17.88%
**miR-128-3p**	3.032	3.198	2.865	46.87%
**miR-451a**	4.430	4.463	4.397	7.92%
**miR-223-3p**	4.709	4.733	4.684	5.83%
**miR-1180-3p**				

Data are indicated as mean Log10(miRNA copy number/μl sample).

Supplementary Table S3: Detection of expression levels of low-abundance miR-186-5p, and miR-1180-3p is highly consistent at different times across multiple samples.

miR-186-5p	Log10			Log10		
	1st Run	2nd Run	3rd Run	Mean	STD	CV
HS1	4.31	4.52	4.46	4.43	0.11	2.50
HS2	3.58	3.85	3.81	3.75	0.15	3.88
HS3	4.09	4.28	4.27	4.21	0.11	2.57
HS4	4.31	4.60	4.48	4.46	0.14	3.24
HS5	4.43	4.63	4.71	4.59	0.14	3.10
**miR-1180-3p**	**Log10**			**Log10**		
	**1st Run**	**2nd Run**	**3rd Run**	**Mean**	**STD**	**CV**
HS1	2.34	2.47	2.49	2.43	0.08	3.42
HS2	2.11	2.18	2.16	2.15	0.04	1.73
HS3	2.28	2.43	2.37	2.36	0.08	3.18
HS4	3.34	3.57	3.43	3.45	0.12	3.42
HS5	3.16	3.31	3.32	3.26	0.09	2.76

Data are indicated as mean Log10(miRNA copy number/µl sample). STD = Standard deviation; CV = Coefficient of variation.

SUPPLEMENTARY FIGURE

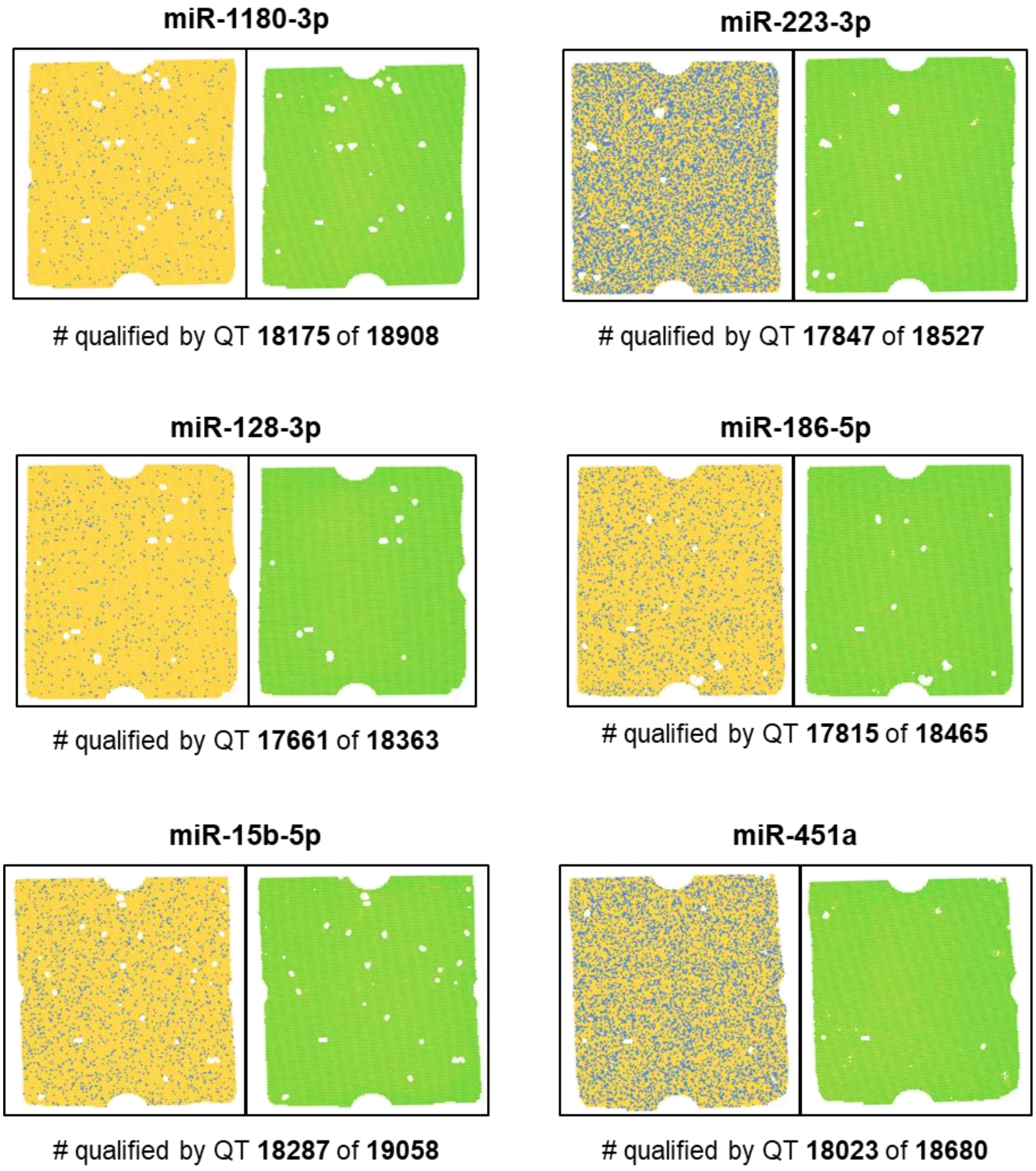

Supplementary Figure S1: miRNA detection in human plasma by digital PCR. The images represent the assay results on > 15.000 nano-wells in the FAM fluorescent channel. The yellow background and dots show the negative nano-wells (without fluorescence), while the blue dots indicate the positive nano-wells (with fluorescence). The green background indicates the goodness of the quality score. # qualified by QT indicates the number of nano-wells with good quality (first number, threshold = 0.6) and the number of all filled nano-wells (second number). The total number of nano-wells per chip is 20000.
